# Flexible time course of spatial frequency use during scene categorization

**DOI:** 10.1038/s41598-021-93252-2

**Published:** 2021-07-07

**Authors:** Sandro L. Wiesmann, Laurent Caplette, Verena Willenbockel, Frédéric Gosselin, Melissa L.-H. Võ

**Affiliations:** 1 Scene Grammar Lab, Department of Psychology, Goethe University Frankfurt, Theodor-W.-Adorno-Platz 6, 60323 Frankfurt am Main, Germany; 2 Department of Psychology, Université de Montréal, Montreal, QC Canada; 3 Department of Psychology, University of Victoria, Victoria, BC Canada

**Keywords:** Psychology, Human behaviour

## Abstract

Human observers can quickly and accurately categorize scenes. This remarkable ability is related to the usage of information at different spatial frequencies (SFs) following a coarse-to-fine pattern: Low SFs, conveying coarse layout information, are thought to be used earlier than high SFs, representing more fine-grained information. Alternatives to this pattern have rarely been considered. Here, we probed all possible SF usage strategies randomly with high resolution in both the SF and time dimensions at two categorization levels. We show that correct basic-level categorizations of indoor scenes are linked to the sampling of relatively high SFs, whereas correct outdoor scene categorizations are predicted by an early use of high SFs and a later use of low SFs (fine-to-coarse pattern of SF usage). Superordinate-level categorizations (indoor vs. outdoor scenes) rely on lower SFs early on, followed by a shift to higher SFs and a subsequent shift back to lower SFs in late stages. In summary, our results show no consistent pattern of SF usage across tasks and only partially replicate the diagnostic SFs found in previous studies. We therefore propose that SF sampling strategies of observers differ with varying stimulus and task characteristics, thus favouring the notion of flexible SF usage.

## Introduction

Despite the visual complexity of the world that surrounds us, the human visual system is usually able to quickly, accurately, and almost effortlessly extract the overall meaning or “gist” of a scene^1^. When, for example, a friend sends us pictures of their new apartment, nothing more than a short glimpse of a room is required to categorize it at the basic level of abstraction, that is, as a kitchen or a bedroom. Superordinate-level categorization, for example, deciding whether a scene is indoor or outdoor, may be achieved even faster^2^. Recent models of visual perception relate this remarkable feat to the fast processing of global image properties in early perceptual stages^3–9^. These low-level image features are thought to quickly convey sufficient information for the categorization of a scene, whereas higher-level features, such as object identity or the semantic and syntactic (i.e., structural) arrangement of objects, are not considered necessary for gist formation (though the identity of some objects may already be apparent after short presentation and processing times^1^).

Besides other visual information like colour^6^ or summary statistics^10^, the ability to quickly categorize scenes has often been linked to the early availability of information at low spatial frequencies (LSFs)^11^. LSFs, which convey information about the coarse shapes contained in a scene, seem to be processed more rapidly by the brain than high spatial frequencies (HSFs) which convey more detailed information^12^ (but see ref.^13^). In addition, previous research indicates that LSFs are used (or attended) before finer information when looking at a scene, which is referred to as a “coarse-to-fine” (CtF) sampling strategy^14–16^ (for results in the object domain see also ref.^17–19^). Observers are faster and more accurate at recognizing scenes when LSF information is presented before HSF information as compared to a presentation of HSFs before LSFs (“fine-to-coarse” sequence; FtC). Bar^3,4,9^ suggests that, during object recognition, LSF information is sampled and processed early on, allowing us to form “initial guesses” about the identities of the target object and the surrounding context (i.e., the scene). HSF information, which conveys finer information (e.g., object contours and details), is used afterwards to refine the hypothesis about the target object’s identity. Importantly, Bar^3,4^ proposes that LSF information is sufficient for the basic-level categorization of a scene (e.g., “beach”) based on behavioral^14^, computational^7^, and neuroimaging evidence^20^.

Despite a considerable number of studies supporting the hypothesis of a temporal precedence of LSF over HSF information during scene perception, there are two caveats to the interpretation of these studies. The first regards the limited temporal resolution of most methods used to study SF usage. Typically, sequences of either low- and high-pass filtered images^16^, hybrid images consisting of the LSF information of one stimulus and HSF information of another^14^, or multiple images filtered with gradually shifting band-pass filters^15^ are presented. Participants are shown to respond faster or more accurately to information presented in a CtF sequence (i.e., first the LSF and then the HSF information of the stimulus) compared to a FtC sequence. While these findings hint at a temporal precedence of LSF usage, the small number of sampled time points (usually 2 to 6) and the small number of considered sequences (usually only CtF and FtC) do not allow drawing conclusions about the *precise* time course of the process and the dynamics that unfold. It remains unclear at what exact point in time HSFs become relevant, whether LSF and HSF sampling are temporally independent or overlapping, and whether SF sampling follows a clear CtF pattern or is non-monotonous (e.g., from low to high and back to low SFs again). Furthermore, better performance for CtF sequences might simply reflect a better capacity of participants to adapt to CtF compared to FtC sequences. Understanding the precise temporal structure of SF usage can have important implications for theories assuming mutual influences of HSF and LSF information extraction and top-down feedback during scene recognition.

A second, similar caveat lies in the limited resolution of most methods in the SF dimension. As pointed out before, most studies assessing SF usage during scene categorization compare large, predefined SF bands. One cannot infer which exact SFs within these bands contribute to scene recognition (e.g., one bigger group of SFs towards the lower end of the band or several smaller groups distributed across the band). Moreover, there are no conventions for the definition of HSFs and LSFs. Cut-off values for filtering are often chosen arbitrarily and therefore are not comparable across studies (see ref.^21^ for a discussion). In the context of scene processing, the cut-offs used to define LSFs vary between 1.1 and 4 cycles per degree (cpd), whereas cut-offs for HSFs range from 3.3 cpd to 6 cpd^16,22^. Consequently, some SFs might be considered LSFs in one study and HSFs in another. Important “intermediate” SFs may even be ignored. Furthermore, the continuous presentation of predefined SF bands can lead to adaptation effects (see above). To our knowledge, only three studies directly assessed the diagnostic SFs for the categorization of real-world scenes by randomly sampling information from the entire SF spectrum of scenes on each trial^23–25^. Taken together, these studies showed that relatively low SFs (< 2 cpd) were essential for the fast categorization of scenes, but that, depending on scene type (indoor vs. outdoor) and categorization level (basic-level vs. superordinate-level categorization), specific higher SFs contributed to fast scene categorization as well^24,25^.

Another important aspect in the discussion of the time course of SF usage is that, while CtF *processing* is thought to be physiologically constrained by the processing speed of the magno- and parvocellular pathways, several authors argue that SF *sampling* (the attending to and usage of information) is flexible and can be influenced by expected stimuli and task constraints^8,26–34^. Previous studies suggested that categorizing scenes at the basic level relied on a wider range of both low and higher SFs (between 1 and 32 cycles per image [cpi]) compared to superordinate-level categorizations (1–15 cpi), but this was mostly due to the basic-level categorization of indoor scenes requiring higher SFs (24–32 cpi) than outdoor scenes (13–15 cpi)^24,25^. A similar pattern can be observed in the comparison of basic- and subordinate-level categorization^29,35^. Based on these findings, we assume that modulating influences like scene features and task constraints may also influence the temporal dynamics of SF sampling.

In summary, the methodological limitations discussed above do not allow for precise conclusions about the exact SFs that drive fast scene categorization and their temporal occurrence. In this study, we overcame these restrictions and, for the first time, assessed the precise time course of SF sampling during basic- (Experiments 1 & 2) and superordinate-level (Experiment 3) scene categorizations. We used a version of the Bubbles technique^36^ adapted to sample information in the SF^37^ and time^18^ dimensions. The Bubbles technique allows assessing the information that participants use to perform a task by randomly sampling the information presented on each trial. In our case, we used short videos in which random SFs of a scene image were revealed at random points in time. We then carried out regression analyses to uncover which SFs presented at which moments led to accurate responses in the categorization task. The advantages of this approach are the random sampling of information on each trial and the high resolution in both the SF and time dimensions without relying on the definition of arbitrary cut-offs for low, high, or bandpass filtering. This ensures that participants do not adapt to specific SF bands that are sampled repeatedly and avoids unnatural sampling strategies since the presented information is unpredictable. Furthermore, the resulting stimuli are more natural in that they cover multiple SF bands at the same time, and a significant portion of the spectrum is shown over the course of a trial. With previous methods, intermediate SFs may be completely missing on all trials, only a small SF band may be shown at any moment, and the SF bands to be shown in later time windows may be predicted from the SF bands shown in early time windows. Lastly, previous studies have demonstrated that our method can uncover an evolving time course of SF usage, with different SF bands used at different moments^18,19^. Using this method, we sought to replicate the diagnostic SFs for scene categorization found in earlier studies and to extend these by taking the time course of SF sampling into account. Furthermore, we investigated the effect of different categorization levels (basic- vs. superordinate-level categorization) on the temporal dynamics of SF sampling.

## General methods

### Overview

We conducted three experiments to assess the time course of SF sampling during the basic-level categorization of *indoor* scenes (Experiment 1), the basic-level categorization of *outdoor* scenes (Experiment 2), and the *superordinate*-level categorization of indoor vs. outdoor scenes (Experiment 3). The experiments were identical except for the different base stimuli used (indoor scenes, outdoor scenes, or both) and varying task constraints (4-alternative forced choice tasks in Experiments 1 and 2, 2-alternative forced choice in Experiment 3, see Procedure).

### Participants

Ninety students participated in the study for course credit or a compensation of €16 (Experiment 1: 24 female, 6 male; 19–53 years old, *M* = 27 years; Experiment 2: 22 female, 8 male; 18–52 years old, *M* = 23 years; Experiment 3: 25 female, 5 male; 18–30 years old, *M* = 22 years). All volunteers were naïve with respect to the hypotheses of the study and were unfamiliar with the images that served as stimuli. We tested the participants’ visual acuity using a Landolt-C chart. All participants gave informed written consent before participating in the study. All three experiments were conducted in accordance with guidelines approved by the Goethe University’s Human Research Ethics Committee. The Goethe University’s Human Research Ethics Committee approved the protocols used in all three experiments.

### Stimuli

**Figure 1:**
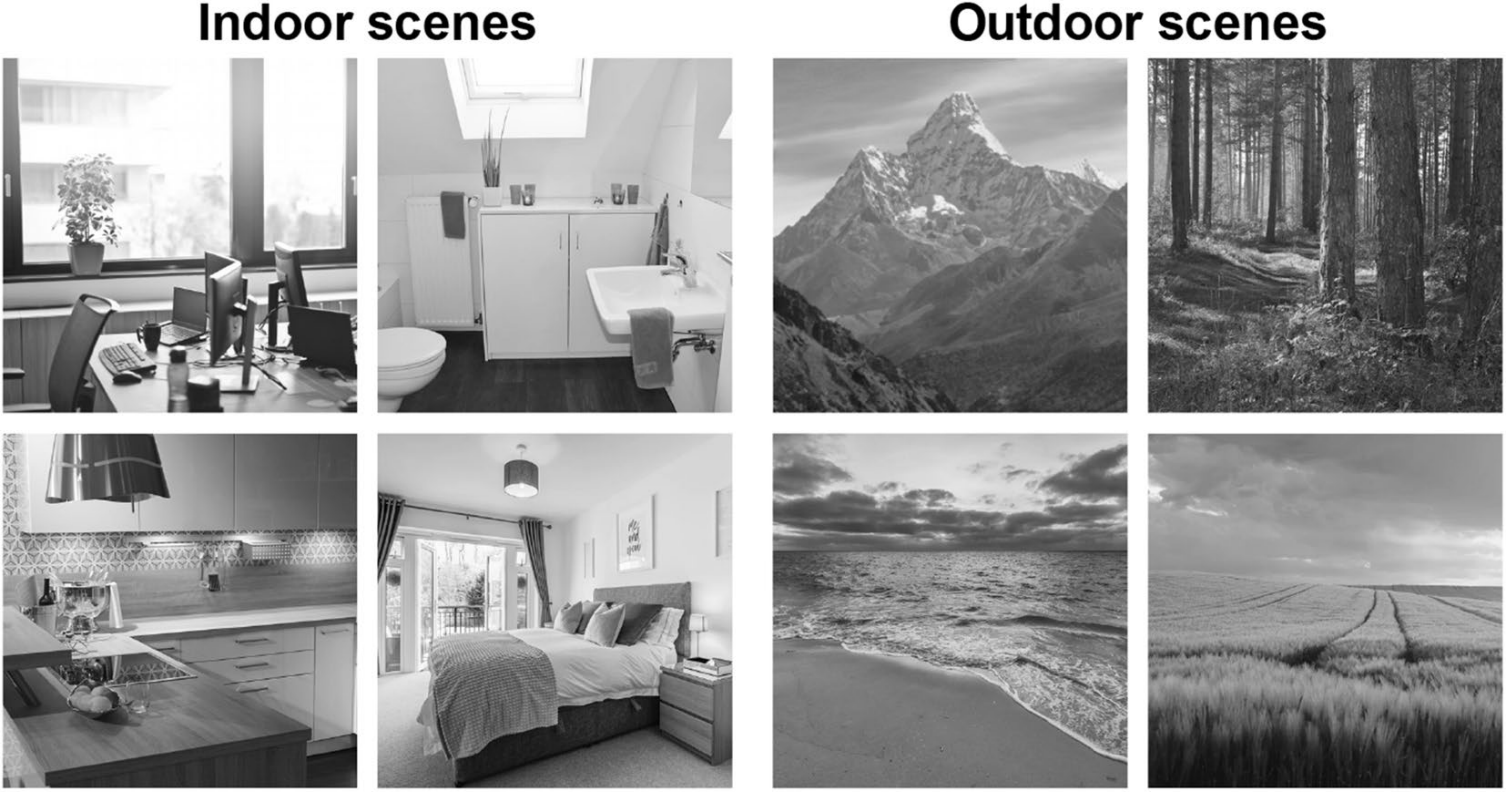
Unfiltered base stimuli. Indoor scenes comprise offices, bathrooms, kitchens and bedrooms. Outdoor scenes comprise mountains, forests, coasts and fields. Images were processed using MATLAB (Version 9.9.0.1495850, https://mathworks.com).

The base stimulus set consisted of 1600 black and white photographs of indoor and natural outdoor scenes (Fig. 1, see also ref.^24,25^). Stimuli could be divided into two superordinate-level categories (indoor and outdoor scenes, 800 stimuli each) and eight basic-level categories (indoor: office, bathroom, kitchen, bedroom; outdoor: mountain, forest, coast, field; 200 stimuli per basic-level category). All stimuli were converted to gray scale, resized, and cropped to 256 × 256 pixels (corresponding to approximately 6 × 6° of visual angle). We matched the luminance histograms of the stimuli for each experiment using the SHINE toolbox^38^ for MATLAB. The RMS contrast of the stimuli was 0.42 in Experiment 1, 0.38 in Experiment 2 and 0.40 in Experiment 3. In previous rating experiments^24,25^, independent raters had performed speeded categorizations of the stimuli and judged their typicality to ensure that all stimuli were typical exemplars of their categories and could be identified easily.

**Figure 2:**
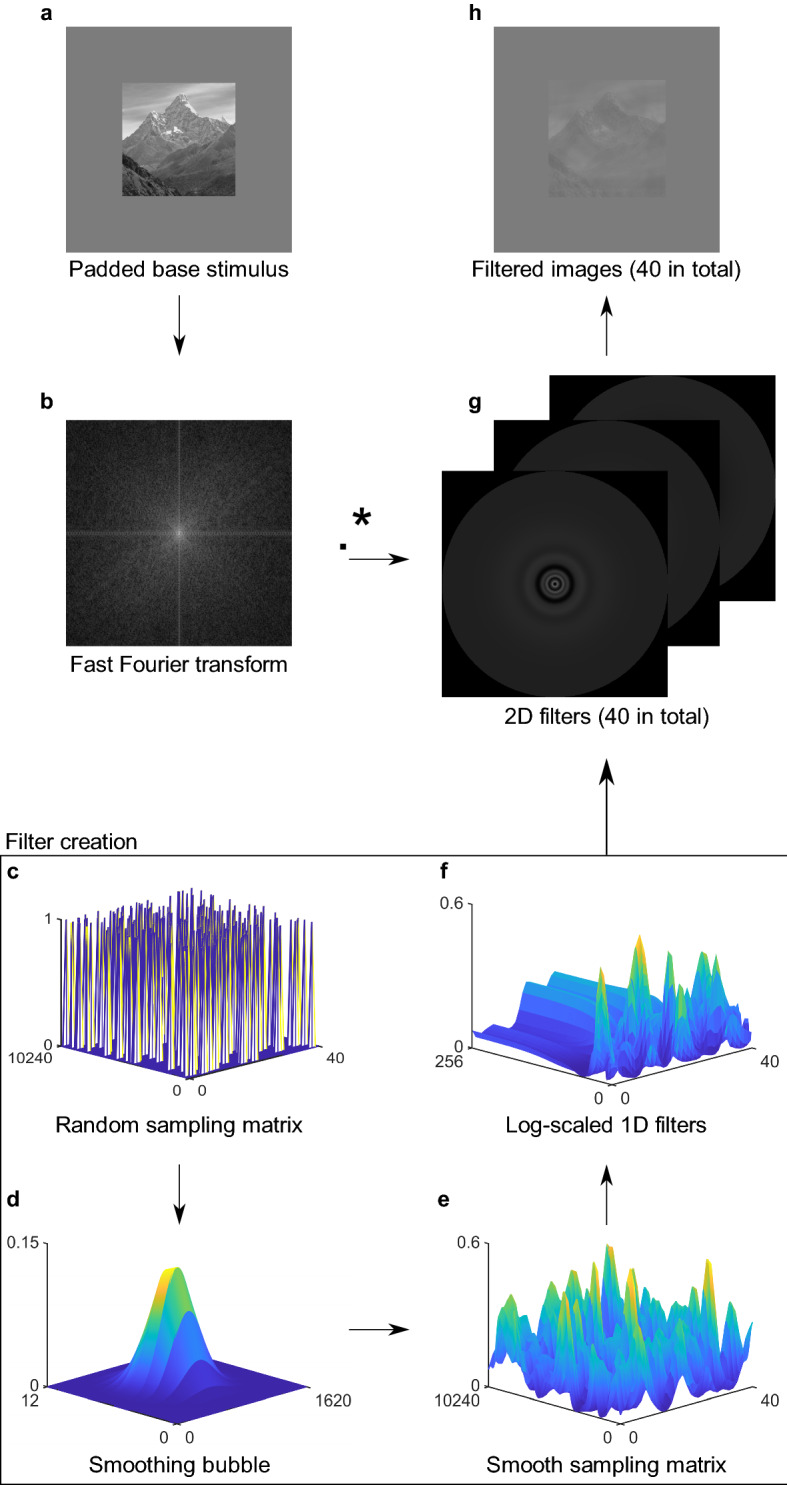
Filtering of stimuli using the SF Bubbles technique (see text for details). (**a**) Padded base stimulus. (**b**) Amplitude spectrum of the padded base stimulus after Fast Fourier transform. (**c**) Random binary sampling matrix. (**d**) Gaussian SF smoothing bubble. (**e**) Smoothed sampling matrix after convolution of the binary sampling matrix with the SF bubble. (**f**) Array of 1D filters after log-scaling of the smooth sampling matrix in the SF dimension. (**g**) 2D filters that are dot-multiplied with the amplitude spectrum of the base stimulus. (**h**) Filtered stimuli after the inverse transformation.

**Figure 3:**
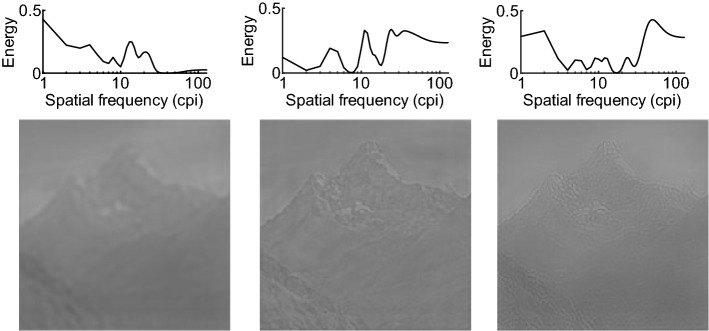
Random 1D SF filters and corresponding filtered stimuli. The filters (upper panel) are created with the SF Bubbles technique (see text for details). Each filter corresponds to one column of the matrix shown in Fig. 2f. The filtered stimuli (lower panel) contain only SF information passing through the corresponding random filters (for the unfiltered base stimulus see Fig. 1). *Note*: In the experiments, we presented short videos consisting of 40 different filtered versions of a stimulus (see Supplementary Videos for examples). cpi = cycles per image.

In the main experiments, we presented short videos (333 ms) consisting of a scene image in which random SFs were gradually revealed at random points in time (see Supplementary Videos for examples). The videos were generated using the SF Bubbles technique^18,37^. We created a new video on each trial as follows (see Fig. 2): First, a base image was randomly selected from the database and padded to avoid filtering artifacts (Fig. 2a). Second, the padded stimulus was fast Fourier transformed (see Fig. 2b for an illustration of the amplitude spectrum of the image). In a third step, we created 40 SF filters to sample information on each of the 40 frames of the video (see Fig. 2c–f). Filters were made from a matrix of size 10,240 × 40 representing the SF × time space. This matrix consisted of mostly zeros and a few ones that were randomly distributed in the matrix using probabilistic sampling (Fig. 2c). The locations of the ones indicate which SFs are sampled at what point in time, whereas SFs set to zero are filtered out of the image. A gradient descent algorithm determined the probability of SFs being sampled on each trial based on the participants’ individual performance to keep accuracy at approximately 75% correct. As a result, the exact number of ones in the matrix varied from trial to trial (Experiment 1: *M* = 180, *SD* = 44; Experiment 2: *M* = 121, *SD* = 39; Experiment 3: *M* = 123, *SD* = 23). To ensure smooth filtering of SFs, we convolved this binary matrix with a Gaussian kernel (maximum = 0.125, σ_SF_ = 270/10,240 elements, σ_Time_ = 2 frames; Fig. 2d). The result of the smoothing is a random distribution of “bubbles” in the SF × time space (Fig. 2e) making sampled SFs gradually appear and disappear over time. The smoothed sampling matrix was further log transformed in the SF dimension to fit the SF sensitivity of the human visual system and to allow for better sampling of lower SF information^39,40^ (Fig. 2f). Each column of this matrix (one for each time frame) was then rotated about its origin to create 40 different 2D filters (Fig. 2g). We then dot-multiplied the stimulus amplitude spectrum (Fig. 2b) with the 40 different 2D filters (Fig. 2g) and subjected the results to inverse fast Fourier transforms (Fig. 2h). After unpadding, the final video consisted of 40 frames (presented for 8.33 ms each) in which random SFs of the base stimulus were gradually revealed over time (see Fig. 3 for examples of random 1D filters and the corresponding filtered stimuli). To ensure accurate luminance display, we applied noisy-bit dithering to the final video^41^.

### Apparatus

The experiment was conducted in a dimly lit booth equipped with a 24-in monitor operating at a resolution of 1920 × 1080 pixels and a refresh rate of 120 Hz. The monitor was calibrated to achieve a linear luminance response. A chin rest was used to maintain a viewing distance of 67 cm. We used MATLAB (MathWorks, Natick, MA) and the Psychophysics Toolbox^42–44^ for stimulus processing, presentation of the experiment, response recording, and statistical analysis.

### Procedure

In Experiments 1 and 2, participants performed a speeded 4-alternative forced choice (4AFC) basic-level categorization task on SF filtered stimulus videos of indoor scenes (bathrooms, offices, kitchens, and bedrooms) and outdoor scenes (mountains, forests, coasts, and fields), respectively. Experiment 3 was identical to Experiment 1 and 2, except participants performed speeded 2-alternative forced choice (2AFC) categorizations of indoor vs. outdoor scenes (superordinate-level categorization). In all experiments, each trial started with the presentation of a fixation cross for 1200 ms, followed by a blank screen with a jittered duration between 300 and 900 ms. A randomly chosen stimulus video was presented afterwards for 333 ms (40 frames, displayed for 8.33 ms each). The stimulus videos consisted of a scene image in which random collections of SFs were revealed at different moments in time (see Stimuli). After stimulus offset, an answer prompt with the possible categories was displayed on the screen until participants indicated the category of the scene using the number keys 1–4 (Experiments 1 & 2) or 1 and 2 (Experiment 3) on the keyboard. We instructed participants to respond as quickly and accurately as possible. After each trial, participants received visual feedback on their response time (RT) and the accuracy of their categorization. The experiment comprised 800 trials (including 20 practice trials) with pauses after each block of 50 trials. Completion of the experiment took approximately 90 min. In Experiments 1 and 2, each participant saw all 800 indoor or outdoor scenes, respectively. In Experiment 3, observers saw 400 indoor and 400 outdoor scenes with 100 scenes from each basic-level category. Stimuli allocations were counterbalanced for pairs of consecutive participants. Half of the stimuli were randomly selected for the first participant from a pair, and the remaining stimuli were allocated to the other participant.

### Analysis

Data from practice trials were discarded. Trials with RTs more than 3 standard deviations from the subject mean were treated as outliers and excluded from analysis (1.25% of trials in Experiment 1, 1.40% in Experiment 2, and 1.26% in Experiment 3). We performed multiple linear regressions between the random SF filters and accuracies of each trial to find out which SFs observers used to correctly categorize a scene and in which time frames these SFs occurred. The analysis was carried out as follows: We first *z* scored accuracies for each participant so that correct trials had positive and incorrect trials negative weights. Afterwards, we normalized all trial-specific binary sampling matrices (that indicate which SFs were sampled at what point in time of each trial, see Fig. 2c), weighted them by the transformed accuracy from their respective trials, and added them up, thus summing the filters from trials leading to correct categorizations and subtracting filters from trials leading to incorrect categorizations (this is equivalent to a multiple linear regression since sampling matrices were random). These raw classification images were then smoothed with a Gaussian kernel (σ_SF_ = 616/10,240 elements, σ_Time_ = 3.74 frames). To establish an empirical null distribution, these analyses were then repeated 1000 times while randomly permuting (with replacement) the accuracies (this disrupts the potential association between accuracies and presentation of SFs). For each observer, the observed classification image was transformed elementwise into *z* scores using the empirical null distribution of classification images. We combined results from all participants and subsampled this *z* map using a logarithmic transformation (see above) to form the group classification images. The pixels of these classification images indicate how the presentation of a given SF on a given video frame correlates with accurate performance in the categorization task on average for the group. To compare SF usage across tasks, we computed the difference between the *z* scored classification images of the tasks and divided by the square root of 2 so that results remain *z* scores. The pixels of these classification images indicate how much more the presentation of a given SF on a given video frame correlates with accurate performance for one task compared to another. We further ran the same analyses on either the SF or time dimension only (collapsing across the other dimension), thus identifying the average importance of SFs across time and the critical time windows for information sampling across SFs, respectively.

Finally, to investigate the time course of SF usage, we estimated linear slopes for each SF across time using the coefficients of the raw classification images. A positive slope indicates that a SF is used increasingly over time whereas a negative slope indicates that a SF is used decreasingly over time. Analyses were repeated on the classification images from the null distribution, and observed slope vectors were *z* scored with vectors of slopes estimated on the null classification images. To compare these temporal trends across tasks, we computed the difference between the *z* scored slope vectors of the tasks and divided by the square root of 2 so that results remain *z* scores.

To keep the family-wise error rate in all analyses at α = 0.05 while performing comparisons over large numbers of SFs and/or time points, we performed cluster tests using the Stat4Ci toolbox^45^. The cluster test identifies groups of adjacent elements in the classification images (or slope vectors) with a *z* score over a critical value (here, *z*_*crit*_ = 3.2). Clusters that exceed a certain number of elements determined by the test are considered significant (i.e., unlikely to have occurred by chance). All tests were two-tailed. Multiple comparisons across tasks were Bonferroni-corrected.

## Results

### Average performance

**Figure 4:**
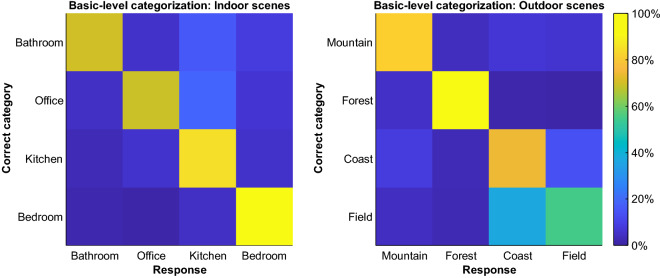
Confusion matrix for the actual category of a stimulus and the participants’ responses in Experiment 1 and 2.

Between-subjects comparisons of RT data from Experiments 1 and 2 revealed that, at the basic level, observers were slower in categorizing indoor scenes (*M* = 883 ms, *SD* = 108) compared to outdoor scenes (*M* = 819 ms, *SD* = 116), *t*(57.75) = 2.19, *p* = 0.03. Furthermore, indoor scenes required more information to be recognized with 75% accuracy (*M* = 0.044%, *SD* = 0.011) than outdoor scenes (*M* = 0.030%, *SD* = 0.010), *t*(57.17) = 5.44, *p* < 0.001. Figure 4 summarizes differences in accuracy between basic categories in Experiments 1 and 2. Of all indoor scenes, both bathroom and office stimuli, the categories with the lowest accuracies, were most often incorrectly categorized as kitchen scenes. Among outdoor scene categories, field scenes were disproportionately often categorized as coast scenes. Between-subjects comparisons of RT data from Experiment 3 with pooled data from Experiments 1 and 2 showed that basic-level categorizations (*M* = 851 ms, *SD* = 116) were carried out more slowly than superordinate-level categorizations (*M* = 670 ms, *SD* = 77), *t*(80.51) = 8.81, *p* < 0.001. Furthermore, participants required more information to reach the 75% accuracy criterion when categorizing scenes at the basic level (*M* = 0.037%, *SD* = 0.013) compared to the superordinate level (*M* = 0.030%, *SD* = 0.006), *t*(87.04) = 3.57, *p* < 0.001.

### Spatial frequency usage

**Figure 5:**
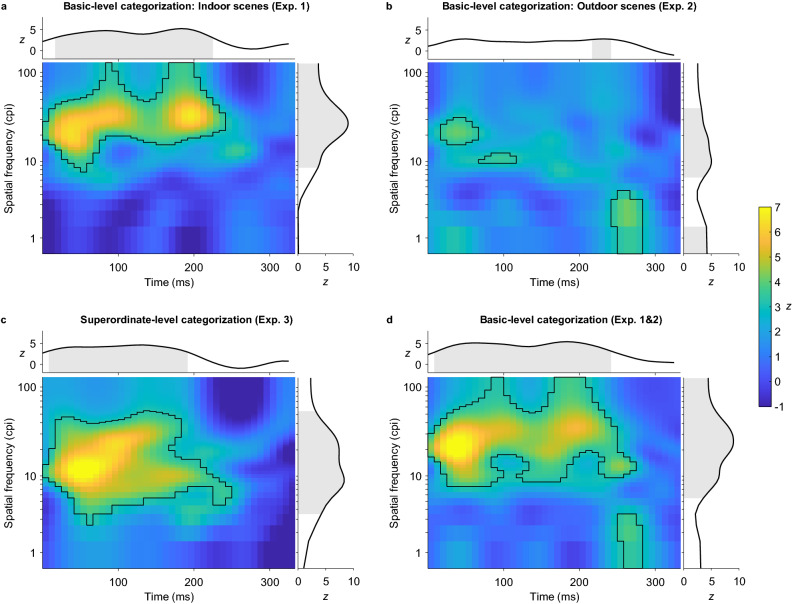
Group classification images obtained from the SF-Bubbles analysis of accuracy data. Basic-level categorization of (**a**) indoor scenes in Experiment 1 and (**b**) outdoor scenes in Experiment 2. (**c**) Superordinate-level categorization of indoor vs. outdoor scenes in Experiment 3. (**d**) Basic-level categorization of indoor and outdoor scenes (pooled data from Experiments 1 and 2). Each pixel indicates how the presentation of a spatial frequency at a given point in time correlates with accuracy in the categorization task. Pixels enclosed by black lines are significant predictors for correct responses. Line plots at the top and on the right of the classification images show results of the Time- and SF-only analyses. Grey areas indicate significant predictors (see text for details). cpi = cycles per image.

Results of the SF × Time analysis of the three experiments are summarized in Fig. 5. The pixels of the classification images indicate how the presentation of a given SF in a given time window correlates with accuracy in the categorization task (brighter colors indicate higher correlations). Black lines enclose clusters of pixels that are significant predictors of correct categorizations (*p* < 0.05, corrected for family-wise error rate). Line plots at the top show significant time points when collapsing data across SFs (time-only analysis). Analogously, line plots on the right show significant SFs when collapsing data across time points (SF-only analysis).

Correct responses in the basic-level indoor scene categorization task (Experiment 1) were predicted by the sampling of SFs between 8 and 128 cpi (1.33–21.33 cpd) spread over a period of 242 ms (0–242 ms; see Fig. 5a). Three peaks occurred within this cluster: The first was centered at 20 cpi and 42 ms (3.33 cpd, *z*_max_ = 6.44), the second at 32 cpi and 92 ms (5.33 cpd, *z*_max_ = 5.93) and the third at 33 cpi and 200 ms (5.50 cpd, *z*_max_ = 6.66). The analysis of SF sampling in the basic-level outdoor scene categorization task (Experiment 2) revealed three smaller clusters of significant SFs (see Fig. 5b). The first cluster ranged from 16 to 31 cpi (2.67–5.17 cpd), spanned 50 ms (17–67 ms) and peaked at 21 cpi and 42 ms (3.50 cpd, *z*_max_ = 4.13). The second cluster included SFs between 10 and 12 cpi (1.67–2.00 cpd), spanned 50 ms (67–117 ms) and peaked at 11 cpi and 100 ms (1.83 cpd, *z*_max_ = 3.60). The third cluster contained SFs between 1 and 4 cpi (0.17–0.66 cpd), spanned 41 ms (242–283 ms) and peaked at 3 cpi and 267 ms (0.50 cpd, *z*_max_ = 4.20). In the superordinate-level categorization task (Experiment 3), correct categorizations were predicted by one larger cluster of SFs between 3 and 54 cpi (0.50–9.00 cpd) spread over a period of 250 ms (0–250 ms; see Fig. 5c). Within this cluster, three peaks occurred: The first was centered at 12 cpi and 58 ms (2.00 cpd, *z*_max_ = 7.58), the second at 22 cpi and 100 ms (3.67 cpd, *z*_max_ = 6.43) and the third at 10 cpi and 167 ms (1.67 cpd, *z*_max_ = 5.09).

**Figure 6:**
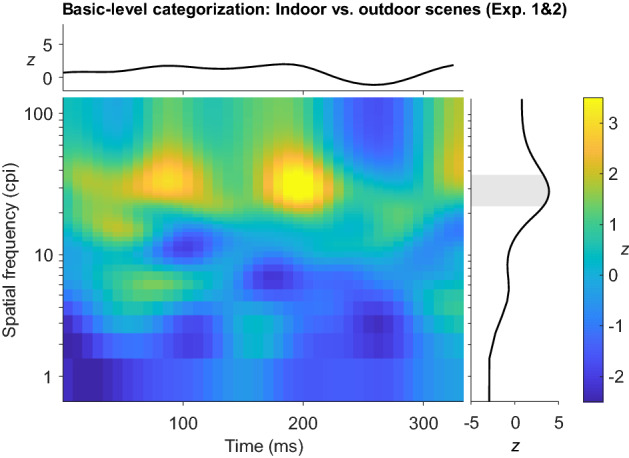
Group classification image obtained for the differences between indoor and outdoor scenes in the SFs sampled across time for the correct basic-level categorization of the respective category. Each pixel indicates how the correlation between the presentation of a spatial frequency at a given point in time and accuracy in the categorization task differs between indoor and outdoor scenes in Experiments 1 and 2 (no significant differences). Line plots at the top and on the right of the classification image show results of the Time- and SF-only analyses. The grey area indicates significant differences between tasks in the sampling of the marked SFs across time. cpi = cycles per image.

Between-subject comparisons of pooled data of Experiments 1 and 2 versus data from Experiment 3 revealed no significant differences in the SF usage between the two categorization levels. Furthermore, SF usage for categorizations at the basic level did not differ significantly between indoor and outdoor scenes at specific time points. These results imply that categorizing scenes from different categories (indoor vs. outdoor stimuli) or at different levels of detail does not significantly influence the usage of single SFs at specific time points. However, when collapsing data across time (SF-only analysis), a band of SFs between 23 and 38 cpi (3.83 and 6.33 cpd; peaking at 29 cpi/4.83 cpd, *z*_max_ = 3.91) was shown to better predict correct basic-level categorizations of indoor than outdoor scenes (see Fig. 6), implying that these SFs were overall more important for indoor than for outdoor stimuli across time points. The Time-only analysis revealed no significant differences between the basic-level categorization of indoor and outdoor scenes or between the basic- and superordinate-level categorization of scenes, indicating that the relevant time windows in which information was sampled were comparable across stimulus categories and categorization levels.

**Figure 7:**
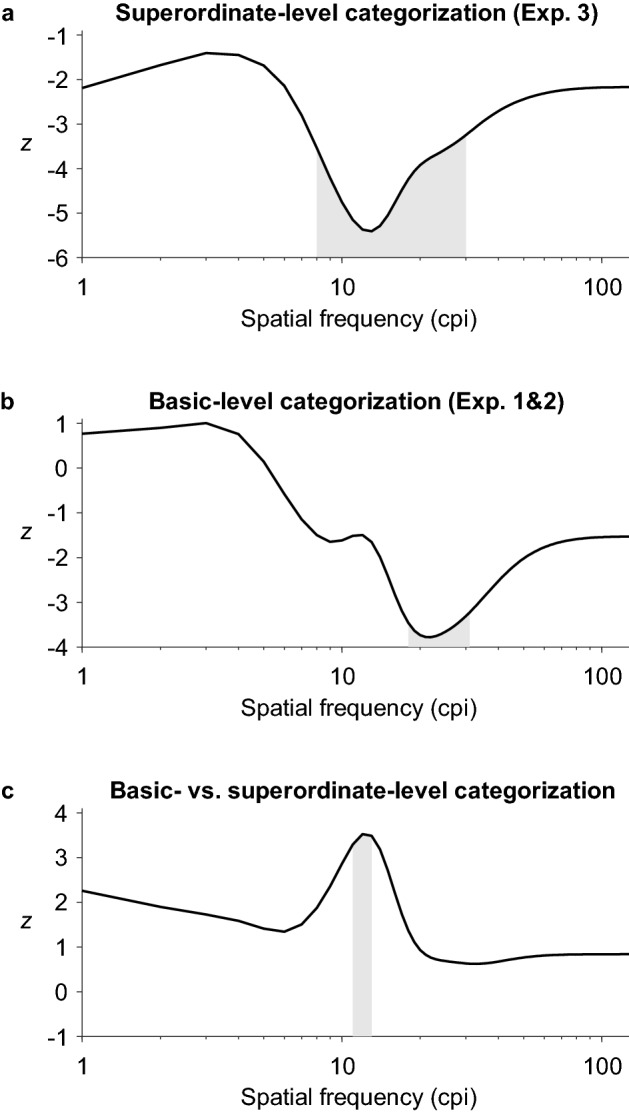
Results of the temporal analysis assessing the time course of SF usage. (**a**) Superordinate-level categorization of indoor vs. outdoor scenes in Experiment 3. (**b**) Basic-level categorization of indoor and outdoor scenes (pooled data from Experiments 1 and 2). (**c**) Differences between basic- and superordinate-level categorization. The *z* values in (**a**) and (**b**) are *z* scored linear slopes indicating the usage of each SF across time (positive values indicate increasing usage across time and negative values indicate decreasing usage across time). The *z* values in (**c**) correspond to the differences between the slopes shown in (**a**) and (**b**). The grey areas indicate slopes (or differences between slopes) that are significantly different from zero. cpi = cycles per image.

The assessment of the time course of SF usage revealed significant linear temporal trends for the superordinate-level categorization (Experiment 3) and basic-level categorization tasks (pooled data from Experiments 1&2). We observed a significantly decreasing usage of SFs between 8 and 30 cpi (1.33 and 5.00 cpd; peaking at 13 cpi/2.17 cpd, *z*_min_ = -5.41) over time for superordinate-level categorizations (Fig. 7a). At the basic level, a band of SFs between 18 and 31 cpi (3.00 and 5.17 cpd; peaking at 22 cpi/3.67 cpd, *z*_min_ = -3.78) was used decreasingly over time (Fig. 7b). Basic- and superordinate-level categorization differed significantly in the usage of SFs between 11 and 13 cpi (1.83 and 2.17 cpd; peaking at 12 cpi/2.00 cpd, *z*_max_ = 3.52) across time: the importance of these SFs decreased more strongly over time in the superordinate- compared to the basic-level categorization task (Fig. 7c). No significant linear temporal trends were observed for the basic-level categorization of indoor (Experiment 1) and outdoor scenes (Experiment 2), respectively, and the comparison of these tasks did not show any significant differences either.

## Discussion

This study assessed the precise time course of SF usage during the basic- and superordinate-level categorization of indoor and natural outdoor scenes. We used an unbiased and high-resolution sampling method based on the SF Bubbles technique^18,36,37^ allowing us to identify the SFs associated with correct categorizations and their exact temporal unfolding. In Experiment 1, we found a large cluster of SFs peaking at 20 cpi/42 ms, 32 cpi/92 ms and 33 cpi/200 ms that predicted correct *basic*-level categorizations of *indoor* scenes (see Fig. 5). In Experiment 2, three small clusters of SFs with peaks at 21 cpi/42 ms, 11 cpi/100 ms and 3 cpi/267 ms predicted correct *basic*-level categorizations of *outdoor* scenes. Experiment 3 once again revealed a larger cluster of diagnostic SFs predicting correct *superordinate*-level categorizations of indoor vs. outdoor scenes that peaked at 12 cpi/58 ms, 22 cpi/100 ms and 10 cpi/167 ms. Our results only partially replicate diagnostic SFs found in earlier studies and reveal varying patterns of SF usage across tasks (CtF, FtC, non-monotonous). Overall, these findings suggest that SF usage across time does not necessarily follow a strict CtF pattern and can be influenced by the stimuli and task design^30^.

One goal of this study was to assess and replicate the precise SFs required for the basic- and superordinate-level categorization of indoor and natural outdoor scenes. The peaks of diagnostic SFs observed here cover intermediate and high SFs for basic-level indoor-scene categorizations (20, 32 and 33 cpi, Experiment 1), intermediate and low SFs for basic-level outdoor-scene categorizations (21, 11 and 3 cpi, Experiment 2) and mostly intermediate SFs for superordinate-level categorizations (12, 22 and 10 cpi, Experiment 3). Note that classifications of low, intermediate and high SFs are arbitrary and depend on the applied definition of cut-offs (see the Introduction of this article and ref.^21^ for a discussion). They are used here to provide a rough reference of how the respective SFs would be typically interpreted. SF usage differed significantly between indoor and outdoor scene categorizations at the basic level (SF-only analysis). SFs between 23 and 38 cpi, which carry rather detailed information about edges and contours in a scene^3,4^, were more predictive of correct indoor compared to outdoor scene categorizations. This could be related to the greater number of small objects and visual clutter typically found in man-made indoor scenes compared to natural outdoor scenes. Observers may have relied more on high SF information related to object identity when distinguishing between a kitchen and a bathroom, for example, whereas low SFs may have been less suitable for this task because of the high similarity of man-made indoor scenes in their general layout^46^.

The trend towards slightly lower SFs for the basic-level categorization of outdoor scenes on the other hand could be due to the large relative size of the main objects or parts of an outdoor scene (e.g., a mountain peak or a tree vs. a computer monitor or a water tap). However, compared to the other two experiments, SF usage during outdoor scene categorization seems to be less distinctive as indicated by overall lower *z* scores and only three small clusters of SFs reaching significance. This may be related to a higher perceptual dissimilarity of outdoor scenes compared to indoor scenes, leading to better categorization performance (as reflected in shorter RTs and a lower amount of revealed information in the stimuli required to reach the accuracy criterion in Experiment 2). As pointed out above, the rooms used here as indoor scenes share many perceptual features (e.g., perspective, depth, etc.) and mostly differ in the semantic assessment of their category-defining objects (e.g., ovens vs. monitors) making high SFs crucial for this task (see also Supplementary Fig. S1). Our outdoor scene categories, on the other hand, have greater variability in basic perceptual features (e.g., depth and openness of a forest vs. a beach). Consequently, there may be less shared information in specific SF bands that is useful for the categorization of, for example, a forest and a beach as compared to a bathroom and an office. SF usage during the categorization of outdoor scenes may therefore be less distinctive because recognition of each category relied on different information (see also Supplementary Fig. S2). Note also that, compared to the SF-only version of the SF Bubbles method, the power of the SF and temporal version used here is lower because the search space extending over 40 frames is considerably larger, making the detection of weaker signals, as in the case of outdoor scenes, more difficult. It is also possible that the less distinctive pattern of SF usage was partly due to participants in this experiment being more variable or noisy; however, we must note that participants from all experiments were recruited in the same way and from the same population.

SF usage at specific time points did not differ significantly between basic- and superordinate-level categorization. However, observers trended toward using a wide range of SFs in the basic-level categorization tasks (all SFs between 1 and 128 cpi except for a small band of SFs between 4 and 7 cpi, see Fig. 5d) whereas they trended toward using a relatively narrow range of SFs in the superordinate-level categorization task (between 3 and 54 cpi, see Fig. 5c). It is conceivable that categorizing scenes at the basic level requires detailed and category-specific information (object-related information conveyed by higher SFs for the categorization of indoor scenes, layout-related information conveyed by lower SFs for the categorization of outdoor scenes). In comparison, superordinate-level categorization (i.e., only deciding whether a scene was indoor or outdoor) is easier and might therefore require fewer detailed information (especially high-SF object-related information), leading to a smaller band of SFs related to correct responses. This is also reflected in the overall lower amount of revealed information in the stimuli required to reach the 75% accuracy criterion (0.037% vs. 0.030% of the search space sampled in basic- vs superordinate-level categorization tasks, respectively).

The general picture of a trend towards higher SFs for the categorization of indoor compared to outdoor scenes at the basic level and a wider range of higher SFs being related to the basic- compared to the superordinate-level categorization of scenes is in line with results of previous studies using the SF Bubbles technique^24,25^. However, the significant SFs we observed in the current study were overall higher than those reported previously and include very few scales typically described as “LSFs”. This is particularly surprising as LSFs are generally suggested to be crucial for fast and accurate scene categorization^3,4,6,9,23^. Previous studies^24,25^ found SFs between 1 and 5 cpi (0.17–0.83 cpd) to be reliably related to fast scene categorization, notably with the same base stimulus set and in all three experimental conditions (indoor and outdoor basic-level categorization and superordinate-level categorization). However, in these studies, stimuli were presented on the screen until participants recognized the scene category (indicated by speeded pressing of the space bar) and only afterwards they indicated the correct category with a second button press. Response times of the space-bar press were then used as the dependent variable of the regression. In the current study, only one button press was required, and categorization accuracy was taken as the dependent variable. We did not analyze RTs here because we were specifically interested in the information used to make accurate categorizations. Moreover, analyzing RTs in relation to temporal usage of information is complicated by the fact that they are partly dependent on one another – small RTs, for example, are likely to be associated only with information presented early on. The presence of diagnostic SFs in lower ranges in previous studies indicates that LSFs are crucial for the *quick recognition* of a scene stimulus, possibly because LSFs are simply processed faster. The current study on the other hand indicates that LSFs are not necessarily related to *correct categorizations*, especially when the task requirements (i.e., the categorization level) afford fine-grained information (as in Experiment 1 for example). It is important to note though that diagnostic LSFs between 0 and 1.74 cpd were reported using a similar method^23^. However, in that study only outdoor stimuli were used (both natural and man-made), and SFs were not filtered out (i.e., their amplitude attenuated), but the phase of non-sampled SFs was replaced with white noise.

The second goal of this study was to assess and compare the precise time course of SF use during the basic- and superordinate-level categorization of real-world scenes. In each of the three experiments, we observed three major peaks that occurred at similar points in time (around 50, 100, and 200 ms) but in different SF ranges. While the peaks of SF usage during the basic-level categorization of indoor scenes roughly followed a CtF sequence over time (20 cpi/42 ms, 32 cpi/92 ms and 33 cpi/200 ms), we observed a FtC pattern with descending SF peaks for outdoor stimuli (21 cpi/42 ms, 11 cpi/100 ms and 3 cpi/267 ms). For superordinate-level categorizations, SF usage showed a non-monotonous pattern over time with peaks in low, higher, and again lower SF ranges (12 cpi/58 ms, 22 cpi/100 ms and 10 cpi/167 ms). However, all three peaks were part of a bigger cluster of significant SFs in which higher SFs were used increasingly over time while lower SFs remained important throughout. Nonetheless, at the superordinate level, usage of SFs between 8 and 30 cpi showed a negative slope across time, indicating that these SFs were more relevant at early frames (i.e., in the first 150 ms, see Fig. 5c) but less so in subsequent ones. Similarly, at the basic level, SFs between 18 and 31 cpi, presumably corresponding to the major peak around 50 ms (see Fig. 5d), were used decreasingly over time. These findings can be interpreted as partial support for the CtF hypothesis: low and intermediate SFs, here under 31 cpi (5.17 cpd), are predominantly used early on and are less relevant in later stages of information sampling. The significant differences in the slopes across time for SFs between 11 and 13 cpi further indicate that the time course of SF usage differs between basic- and superordinate-level categorizations.

Overall, we found CtF-like patterns of SF usage in two of our three experiments. However, as pointed out before, most of the significant SFs reported here do not belong to ranges typically described as “LSFs” and therefore impede any conclusions about the CtF hypothesis as it is typically put forward^3–5^. Moreover, we observed a FtC sampling strategy during the basic-level categorization of outdoor scenes which further challenges the notion of a default CtF usage of information. Interestingly, the pattern of SF usage in this experiment was mostly driven by mountain scenes (see Supplementary Fig. S2). SF usage during the categorization of other outdoor scenes was less distinctive which may be related to the high level of confusions between field and coast stimuli (see Fig. 4) and the varying size of tree trunks in forest scenes as a function of the distance to the photographer. Possibly, as opposed to other categorization tasks, the best strategy for the categorization of outdoor scenes was to look for specific diagnostic HSF features first (e.g., jagged edges of the mountain scenes that were easy to recognize). Only afterwards, LSFs became relevant either to confirm this first guess or to flesh out the spatial layout of the stimulus if no feature allowed an unambiguous categorization. Participants reporting to look for “dead giveaways” such as mountain peaks or palm trees support this assumption.

We believe that the lack of a clear pattern of SF usage over time supports the hypothesis that information sampling under varying task conditions is flexible^30,34^. This is further underlined by our findings of different diagnostic SFs for the basic-level categorization of indoor and outdoor scenes (SF only analysis) and varying patterns of SF usage across time between basic- and superordinate-level categorizations (slope analysis). However, it is important to note that, while we do find mixed evidence for CtF *sampling*, our results do not preclude CtF *processing* of information (i.e., LSFs being processed faster than HSFs by the brain). Assessing information usage at the behavioral level does not provide direct insights into the mechanisms of SF processing at the neural level (e.g., processing of different SFs through the magno- and parvocellular pathways^28^).

We further cannot determine whether the differences in the SFs used and the order they are sampled in are the effect of “bottom-up” or “top-down” processes. On the one hand, the information available in the different image categories restricts the SFs sampled by the observer (e.g., the predominance of higher SFs in indoor scenes may bias the observer to use these SFs). On the other hand, advance knowledge of the categorization level in the task may determine top-down SF sampling strategies that observers use (e.g., knowing that one has to distinguish between indoor and outdoor scenes creates a bias towards lower SFs based on previous experience). This knowledge may also be developed or strengthened during the task by repeatedly categorizing scenes from the same two or four different target categories. On the one hand, a fixed set of possible categories to choose from more closely resembles the assumptions we have about the possible scenes we may encounter in the real world (e.g., in a house or, more specifically, a residential vs. a public building). On the other hand, it may have reinforced the adaptation of information sampling strategies tailored to distinguish between the specific scene categories that we selected in our tasks (see discussion of SF usage for outdoor scenes above).

On a methodological level, our results underline the necessity of high-resolution sampling methods to uncover the precise usage of information in a task. Conventional low-, high-, or bandpass filtering designs may not be sufficient to grasp the whole story of SF usage and its temporal unfolding. In our experiments, we found a variety of diagnostic SFs that could be considered low or high SFs depending on the definition applied or that fall in an “intermediate” range of SFs not even considered by some other authors. Results from studies using arbitrary filter cut-offs are not only difficult to compare with each other but may also be imprecise when important SFs are omitted. The same is true for the assessment of the time course of information usage. While overall linear trends of SF sampling (such as CtF or FtC strategies) might be robust enough to be captured when temporal resolution is reduced to the sampling of a few time points, finer differences in the temporal unfolding of information usage or non-linear trends cannot be discovered.

In summary, our findings do not support the notion of a default CtF information sampling strategy during the categorization of real-world scenes. We observe CtF, FtC, and non-monotonous patterns of SF usage depending on the stimulus set (indoor vs. outdoor scenes) and the categorization level (basic vs. superordinate), thus favouring the notion of flexible information usage during scene categorizations under varying task conditions. Whether these differences reflect bottom-up differences in the perceptual features best suited to distinguish between the categories or top-down influences in the form of SF-sampling strategies adjusted to the task requirements remains unclear. Either way, the human visual system seems to flexibly adapt to varying task demands and efficiently use specific information suited to reach the current goal of the observer.

## Supplementary Information

Supplementary Video 1.

Supplementary Video 2.

Supplementary Information 1.

## Data Availability

The datasets generated during the current study and computer code used for data analysis are available from the corresponding author upon request.
