# Supplementary material for: Flexible time course of spatial frequency use during scene categorization
Source: Sci Rep. 2021 Jul 7;11:14079. doi: 10.1038/s41598-021-93252-2 (PMC8263560; doi:10.1038/s41598-021-93252-2)
Supplement: Supplementary file 3 — Supplementary Information 1. [file 41598_2021_93252_MOESM3_ESM.pdf]

Flexible Time Course of Spatial Frequency Use During Scene Categorization

Sandro L. Wiesmann[1*], Laurent Caplette[2], Verena Willenbockel[1,3],

Frédéric Gosselin[2], and Melissa L.-H. Võ[1]

[1]Department of Psychology, Johann Wolfgang Goethe-Universität, Frankfurt, Germany

[2]Department of Psychology, Université de Montréal, Montréal, QC, Canada

[3]Department of Psychology, University of Victoria, Victoria, BC, Canada

*Corresponding author. Email: wiesmann@psych.uni-frankfurt.de.

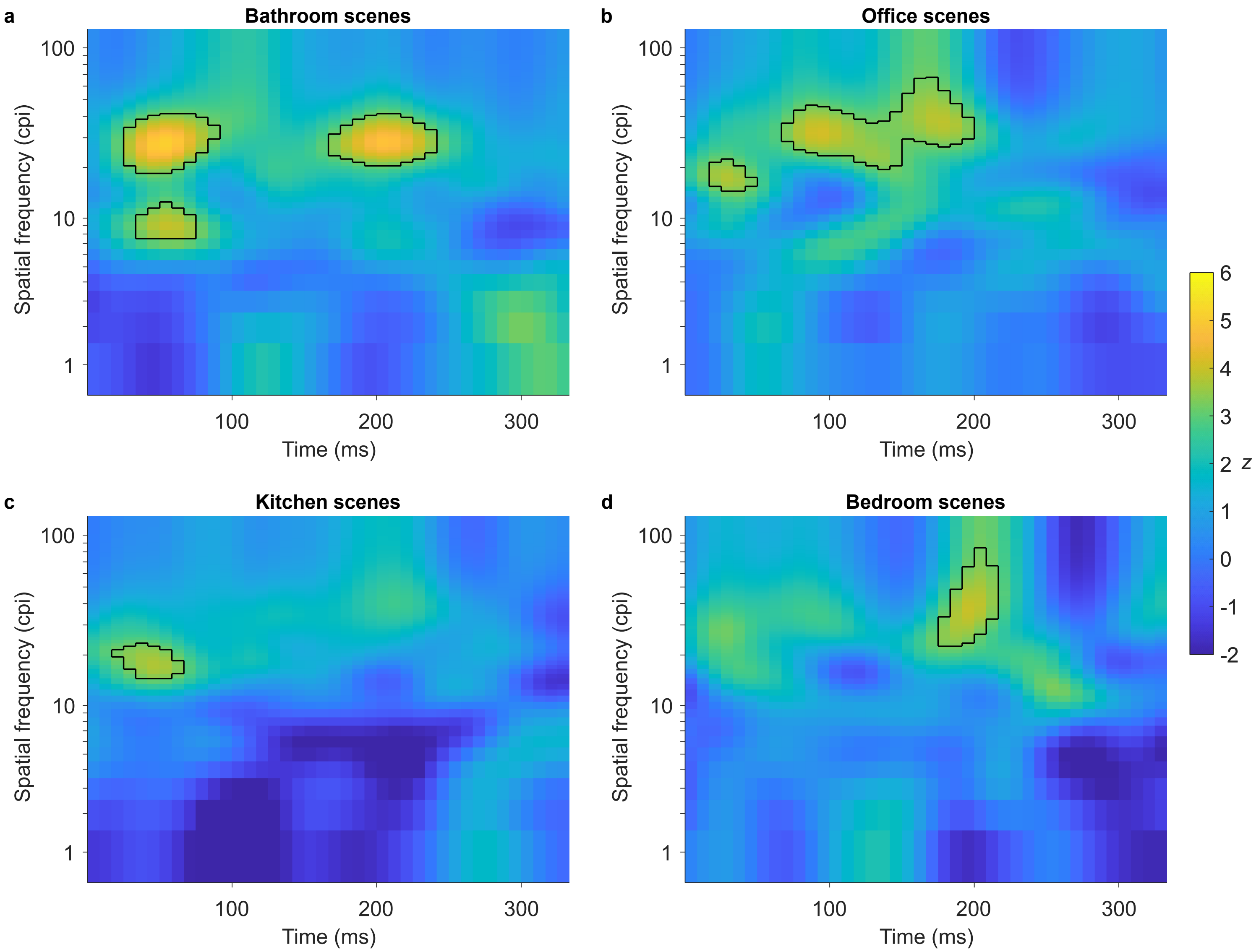

Supplementary Figure S1. Group classification images obtained from the SF-Bubbles analysis of accuracy data in Experiment 1 for subsets of (a) bathroom, (b) office, (c) kitchen, and (d) bedroom scenes. Each pixel indicates how the presentation of a spatial frequency at a given time point correlates with accuracy in the categorization task. Pixels enclosed by black lines are significant predictors for correct responses. cpi = cycles per image.

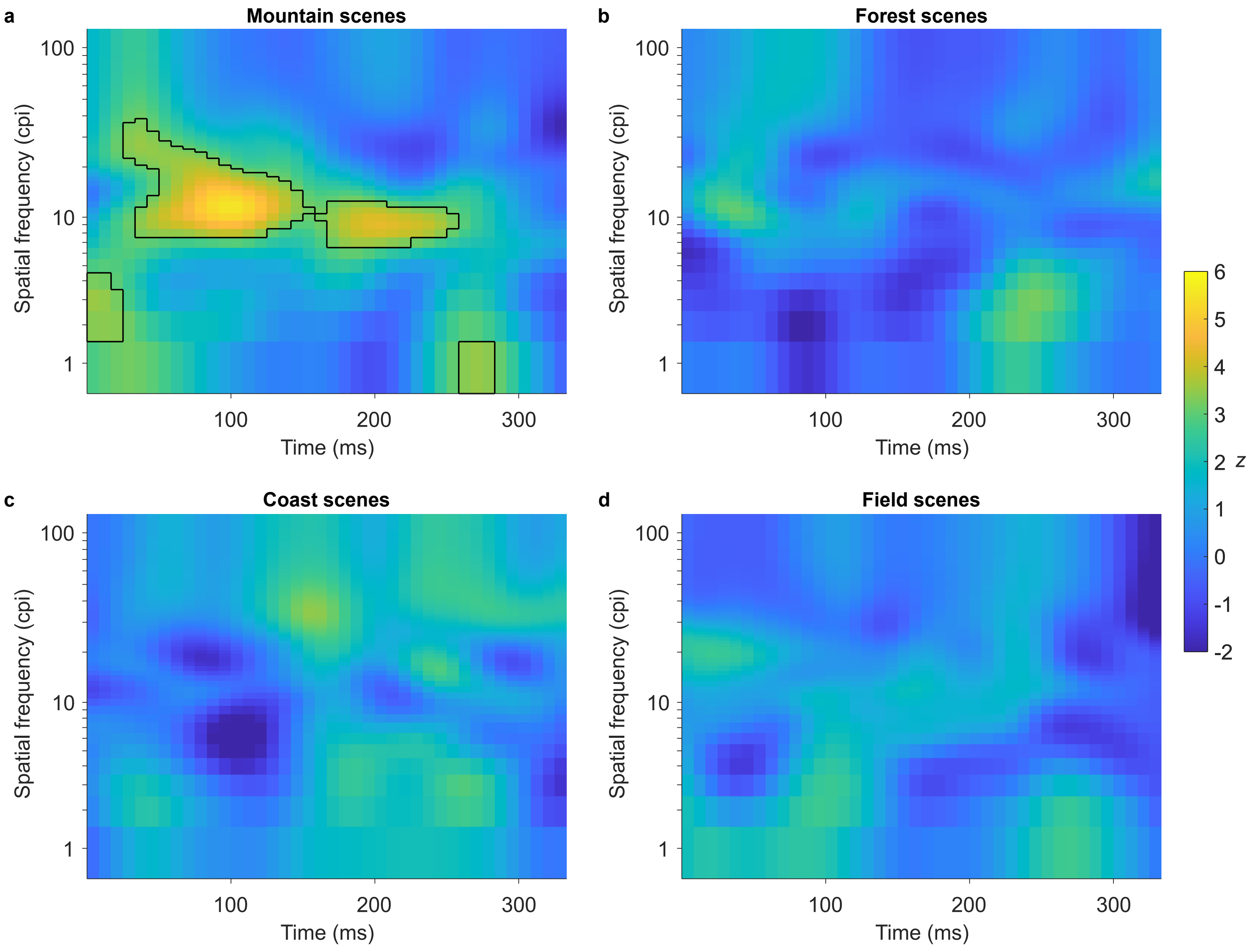

Supplementary Figure S2. Group classification images obtained from the SF-Bubbles analysis of accuracy data in Experiment 2 for subsets of (a) mountain, (b) forest, (c) coast, and (d) field scenes. Each pixel indicates how the presentation of a spatial frequency at a given time point correlates with accuracy in the categorization task. Pixels enclosed by black lines are significant predictors for correct responses. cpi = cycles per image.

Supplementary Video S1. Example of a stimulus video used in the experiments. Random SFs are revealed at random time points.

Supplementary Video S2. Example of a stimulus video used in the experiments (slowed down to a quarter of the original speed for illustration). Random SFs are revealed at random time points.
